# Targeting aryl hydrocarbon receptor to prevent cancer in barrier organs

**DOI:** 10.1016/j.bcp.2024.116156

**Published:** 2024-03-20

**Authors:** Francoise Congues, Pengcheng Wang, Joshua Lee, Daphne Lin, Ayaz Shahid, Jianming Xie, Ying Huang

**Affiliations:** a Department of Biotechnology and Pharmaceutical Sciences, College of Pharmacy, Western University of Health Sciences, Pomona, CA 91766, USA; b Department of Stomatology, Beijing Chao-Yang Hospital, Capital Medical University, Beijing, China

**Keywords:** Aryl hydrocarbon receptor, Polycyclic aromatic hydrocarbons, Cancer, Carcinogenesis, Skin, Lung, Colon

## Abstract

The skin, lung, and gut are important barrier organs that control how the body reacts to environmental stressors such as ultraviolet (UV) radiation, air pollutants, dietary components, and microorganisms. The aryl hydrocarbon receptor (AhR) is a ligand-dependent transcription factor that plays an important role in maintaining homeostasis of barrier organs. AhR was initially discovered as a receptor for environmental chemical carcinogens such as polycyclic aromatic hydrocarbons (PAHs). Activation of AhR pathways by PAHs leads to increased DNA damage and mutations which ultimately lead to carcinogenesis. Ongoing evidence reveals an ever-expanding role of AhR. Recently, AhR has been linked to immune systems by the interaction with the development of natural killer (NK) cells, regulatory T (T_reg_) cells, and T helper 17 (Th17) cells, as well as the production of immunosuppressive cytokines. However, the role of AhR in carcinogenesis is not as straightforward as we initially thought. Although AhR activation has been shown to promote carcinogenesis in some studies, others suggest that it may act as a tumor suppressor. In this review, we aim to explore the role of AhR in the development of cancer that originates from barrier organs. We also examined the preclinical efficacy data of AhR agonists and antagonists on carcinogenesis to determine whether AhR modulation can be a viable option for cancer chemoprevention.

## 1. Introduction

Barrier tissues, including skin, airway, gastrointestinal tract, and urogenital tract, play an essential role in maintaining local and systemic homeostasis [1]. Particularly, the skin, lungs and gut are important barrier organs that determine how the body reacts to external stressors, including ultraviolet (UV) radiation, pollutants and microbiota in the air and food. These organs share similarities in responses to environmental insults by orchestrating common signal transduction pathways [1,2]. The skin, lung and gut also serve as immune organs since a wide variety of innate and adaptive immune reactions can occur at these sites. Disturbed barrier homeostasis leads to a variety of diseases, including cancer. Aryl hydrocarbon receptor (AhR) is a fascinating molecule for cancer researchers who focus their work on barrier organs, since AhR is highly expressed in those tissues which are the primary sites of contact with ligands of AhR [3]. The AhR ligands in the environment can be both beneficial and harmful. Some AhR ligands, including the polycyclic aromatic hydrocarbons (PAHs) such as benzo(a)pyrene (BaP), can induce carcinogenesis of lung and skin. Some other AhR ligands, such as plant-occurring flavonoids, can be cancer preventive [4].

**Fig. 1.**
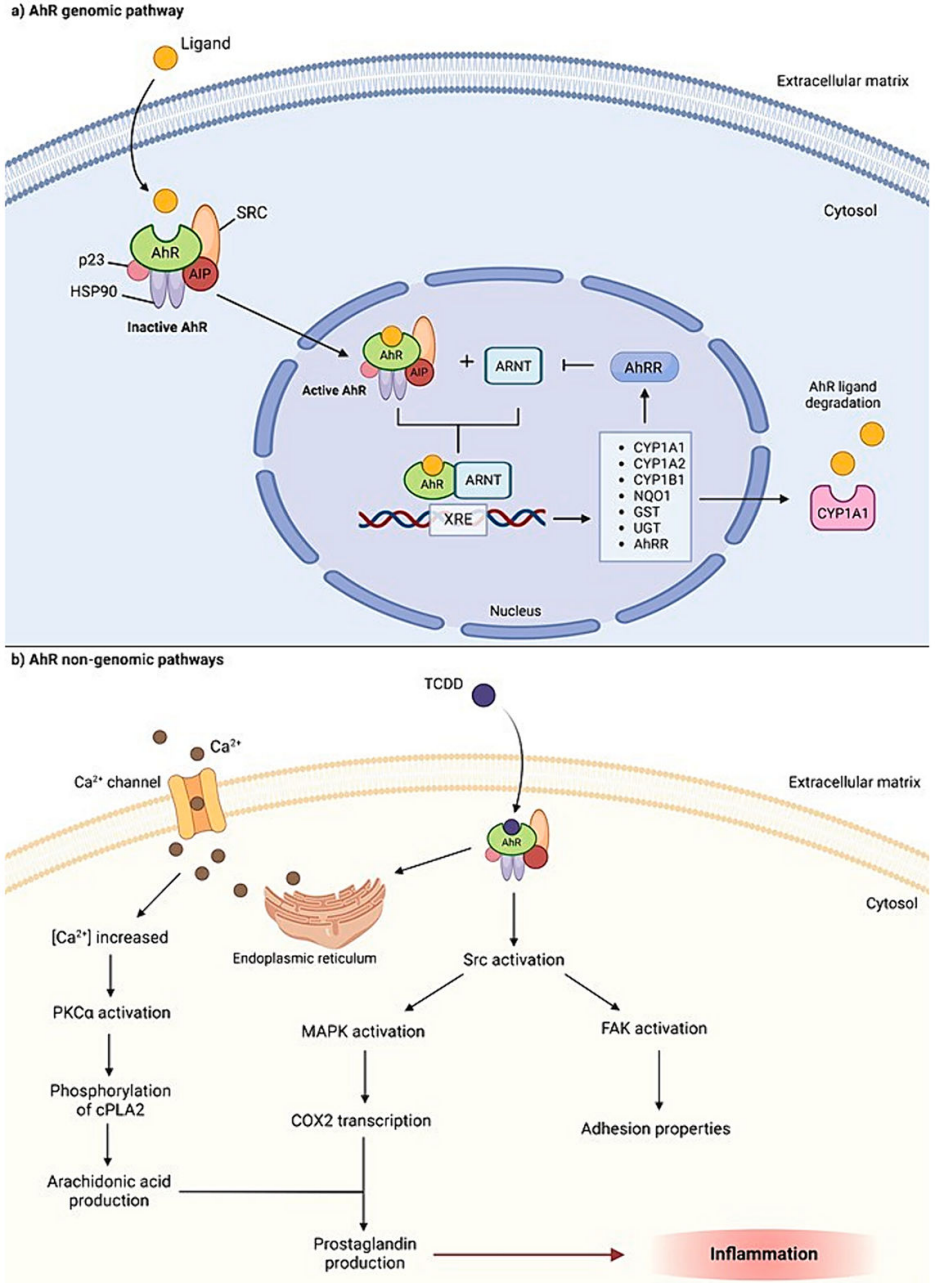
Outlines of the AhR signaling pathway. a) Inactive AhR is cytosolic and forms a complex together with chaperone proteins HSP90, Src, AIP, and p23. Binding between AhR and its ligand translocates the activated ligand-AhR complex into the nucleus, where it dissociates from its chaperone proteins. The complex heterodimerizes with AhR Nuclear Translocator (ARNT) and binds to Xenobiotic-Responsive Elements (XRE) in the promoter regions of target genes [173], enabling modulation of target gene expression by inducing mRNA transcription. Some target genes include CYP1A1, CYP1A2, CYP1B1, NAD(P)H quinone dehydrogenase 1 (NQO1), glutathione s-transferase (GST), UDP-Glucuronosyltransferase (UGT), and aryl hydrocarbon receptor repressor (AhRR). AhRR suppresses AhR activity through binding with ARNT and XRE [174-176]. b) Following exposure to a highly carcinogenic ligand, like 2,3,7,8-tetrachlorodibenzo-p-dioxin (TCDD), intracellular calcium concentrations increase, sourced extracellularly and from the endoplasmic reticulum [173]. TCDD induces functional activation of tyrosine kinase Src which is released from the AhR complex [177], altering cellular adhesion properties in the process by activating focal adhesion kinase (FAK) [177]. Calcium influx activates protein kinase C (PKCα) which phosphorylates phospholipase A2 (cPLA2), stimulating the production of arachidonic acid [173]. Src activation also activates mitogen-activated protein kinases (MAPK), ERK-1 and –2, allowing the transcription of cyclooxygenase-2 (COX2) which utilizes arachidonic acid to produce prostaglandins, ultimately modifying pathophysiological process like inflammation [173,178]. (Created with BioRender.com).

AhR is a transcription factor that belongs to the basic helix-loop-helix-PER-ARNT-SIM (bHLH-PAS) superfamily [5] and was initially discovered as an environmental sensor for external chemicals [6]. For several decades, it has been confirmed that AhR plays a role in mediating the toxic effects of environmental carcinogens that are present in cigarette smoke and air pollution. The AhR pathway can be activated after binding to a wide range of chemicals that are either natural, like B(a)P, or synthetic, like 2,3,7,8-tetrachlorodibenzodioxin (TCDD), two ubiquitous AhR ligands with notoriously high carcinogenicity. Depending on the ligand, one of several AhR pathways can be activated which participate in carcinogenesis, either genomic or non-genomic (Fig. 1). Therefore, AhR has become a pathway of interest for researchers in the areas of toxicology and carcinogenesis since the 1990s [7]. Recently, AhR has been recognized with functions beyond its role as a sensor for TCDD and PAHs. For example, the tryptophan (Trp) catabolite kynurenine (Kyn) was found as an endogenous AhR ligand that can bind to and activate AhR, and upregulate the expression of growth-controlling genes in the colon [8] and lung cancer cells [9]. Kyn-activated AhR induces immunosuppressive effects and creates a microenvironment that is defective in organizing to eliminate cancer cells [10]. Since AhR plays an important role in immunology, soon it became the pathway of interest for the immunology community [7]. Due to the importance of the immune system in carcinogenesis, the AhR pathway must play a significant role in cancer development. Therefore, the eruption of studies concerning the role of AhR in immunology and carcinogenesis were imperative .

However, the role of AhR in cancer is not as straightforward as we initially understood. Although some studies provide evidence that activation of AhR pathway promotes carcinogenesis, others suggest an opposite function of AhR as a tumor suppressor. The question remains unanswered as to whether AhR is a target for preventing carcinogenesis, particularly for cancers originating from barrier organs. This information is essential before screening, developing, and applying AhR modulators for cancer chemoprevention. This review aims to discuss AhR’s contribution to the development of the common types of cancer that originate from three main barrier organs, including the skin, lungs, and gut, and to review the efficacy of AhR agonists and antagonists on preclinical carcinogenesis models, and address the question as to whether or not AhR modulation can be used for cancer chemoprevention.

## 2. Genetic evidence for the role of AhR in carcinogenesis

Genetic and genomic studies have demonstrated a possible role of AhR in cancer development, where it can act as a positive or negative regulator for carcinogenesis. By analyzing the genetic data of AhR in cancer using available genomic data, for example, cBioPortal, in a searchable online genomics platform (https://cbioportal.org), only a very small proportion of amplification, deletion, or mutation in the AhR gene was found [11]. Interestingly, AhR somatic mutations have been specifically detected in samples from urinary tract cancer, primarily bladder cancer. The non-synonymous mutation Q383H has been detected in bladder cancer, and predicted as an activating mutation, and associated with higher AhR mRNA expression and activity [12]. In addition, it was found that the recurrent in-frame deletions of exons 8 and 9 in the AhR gene displayed a high prevalence in bladder cancer comprising ~ 10 % of patients, which is highly specific to urinary tract cancer and mutually exclusive with other bladder cancer drivers [13]. Functional studies have shown that these AhR somatic mutations activate the canonical AhR signaling and downstream gene expression: the Q383H mutation leads to more sensitized ligand-dependent activation, while the in-frame deletion of exons 8 and 9 leads to ligand-independent constitutive activation of the AhR pathway, promoting bladder cell transformation [13]. Thus, activating mutations in AhR could be a driving event for bladder cancer. Patients who harbor AhR-activating mutations may benefit from AhR-targeted therapies [14], although it is unknown why AhR somatic mutations are specific to bladder cancer.

Despite AhR not having recurrent genetic abnormalities in most cancer types, its expression is often altered in various types of cancer. For example, the mRNA or protein of AhR and its downstream genes were found overexpressed in breast cancer [15-17], non-small cell lung cancer [18], thyroid cancer [19,20], cutaneous squamous-cell carcinoma [21], oral squamous cell carcinoma [22], pancreatic cancer [23], endometrial cancer [24], and meningioma [25]. AhR expression was elevated in the early stages of cancer regardless of cancer type, suggesting that AhR is involved in early events in most cancers. Nuclear localization of AhR was correlated with the worst outcomes for patients with non-small cell lung cancer [18,26]. Furthermore, not only the malignant cells, but also the cells within the tumor microenvironment express high levels of AhR [16].

Genetic evidence suggests that AhR can inhibit carcinogenesis. For example, the AhR mRNA expression was downregulated in primary peripheral blood chronic myeloid leukemia (CML) cells compared to the healthy controls [27]. In this study, AhR antagonists induced expansion of leukemic cells, while AhR agonists reduced the colony-forming cells in CML. These data supported the cell-specific functions of AhR. For lung cancer, immunohistochemistry studies on human tissue samples revealed that normal human lung tissues expressed high levels of AhR. In contrast, AhR expression was much lower in the lung cancer tissues, including samples of lung squamous cell carcinoma, lung adenocarcinoma and invasive lung cancer. Furthermore, AhR expression was diminished in the metastatic lung tumors, suggesting that AhR may have tumor suppressor-like activity for human lung cancer [28]. In addition, the cancer patient survival data showed that a high level of AhR in cancers of the lung, breast, esophagus, kidney, and uterus predicts better overall survival of the patients, whereas the survival probability of lower AhR-expressing cancers is significantly poorer. It is still unknown why different studies produced different results in these types of cancer. The tumor-suppressive function of AhR has been reviewed recently [29].

AhR can also regulate the expression of genes involved in several other pathways. For example, the DNA methylation mechanism contributes towards the epigenetic regulation of multiple biological processes such as transcriptional silencing of tumor suppressor genes in cancers [30,31] and marking transcriptional start sites as well as the boundaries between introns and exons in transcribed genes [30,32,33]. Notably, the inducibility of CYP1 (cytochrome P450, family 1) by ligands of AhR can be enhanced by DNA methylation following treatment with DNA methyltransferase inhibitors [34,35]. There exist eight putative XRE sites that can allow binding of ligand-activated AhR within the CYP1B1 gene’s enhancer regions and because each XRE sequence contains a CG site that can undergo reversible methylation, this means that DNA methylation can impact CYP1B1 inducibility as methyl groups in CG sites can directly hinder the binding of AhR to XRE sequences [30,36]. Another important process influenced by AhR activation is *N*^6^-methyladenosine (m6A) RNA methylation, a common site for post-transcriptional RNA modification [37]. One study demonstrated the effects of developmental exposure of m6A methylation patterns to PCB126, a dioxin-like AhR ligand belonging to the polychlorinated biphenyl (PCB) class [37]. Using RNA immunoprecipitation and sequencing, higher incidences of transcript methylation were found, which can be activated by AhR like aryl hydrocarbon receptor repressor (AHRR), in response to PCB126 than to DMSO, suggesting that by altering m6A levels, xenobiotics like PCBs could affect developmental gene expression patterns [37]. Despite there being several main types of epigenetic modifications induced by environmental toxicants like DNA methylation, histone alterations, and non-coding RNA, m6A modifications in mRNA are arguably the most notable, given the accumulating evidence suggesting its wide involvement in pathological process at the molecular and cellular level [38,39]. It was recently reported that abnormal m6A levels, which can result from environmental exposure to PAHs, have associations with the malignant progression in multiple cancers such as lung adenocarcinoma, acute myeloid leukemia, ovarian cancer, hepatocellular carcinoma, gastric cancer, colorectal cancer, and melanoma [38,40]. The associations attributed to m6A in these cancers include proliferation, invasion, metastasis, and drug resistance as the proteins that regulate m6A RNA methylation are able to recognize both oncogenes and tumor suppressor genes [38,41,42]. The involvement of AhR in this array of pathways sheds light on the multifaceted mechanisms through which AhR contributes to the development of cancer.

## 3. Functional studies of AhR in carcinogenesis induced by environmental factors

The function of the AhR in carcinogenesis induced by environmental factors has been investigated by RNA interference, gene overexpression, pharmacological interference, and rodent genetic models. The role of AhR in carcinogenesis of skin, lung, and gut, mediated by the most common carcinogens, is briefly summarized here.

### 3.1. AhR in UV-associated skin carcinogenesis

Skin is a barrier providing vital protection against physical or chemical harm as well as infection [43]. Skin cancer includes nonmelanoma skin cancer (NMSC) and melanoma. NMSC, encompassing basal cell carcinoma (BCC) and squamous cell carcinoma (SCC), represents the most common type of cancer [44]. Malignant melanoma, although constituting less than 5 % of all diagnosed skin cancers, is responsible for the majority of skin cancer-related deaths due to its aggressive metastatic nature [44]. Repeated and unprotected exposure to UV radiation, particularly the shorter wavelength ultraviolet B (UVB) component, is the primary risk factor for developing most types of skin cancer in the general population. Although the mechanisms are not fully understood, UVB triggers DNA damage, skin inflammation and immunosuppression, which altogether contribute to skin carcinogenesis.

**Fig. 2.**
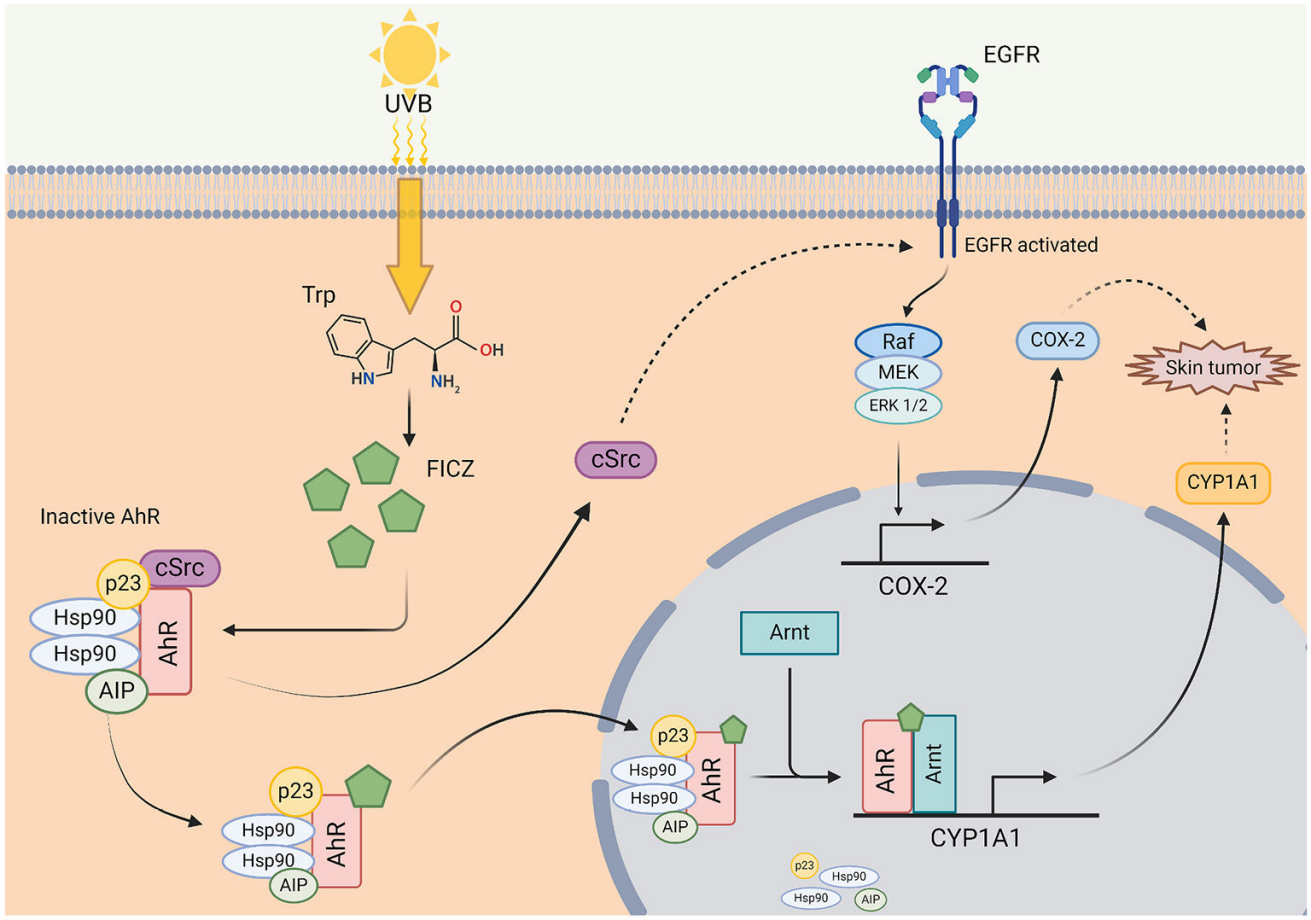
FICZ-induced, AhR-mediated skin carcinogenesis from UV radiation. FICZ (6-formylindolo[3,2-b]carbazole) is a tryptophan photoproduct in the skin, derived upon UVB exposure. FICZ is a high-affinity AhR agonist in which binding will trigger the canonical AhR pathway thus upregulating the expression of AhR target genes such as CYP1A1 [179]. When FICZ binds to the cytosolic AhR complex, cSrc is activated and dissociates from the complex. cSrc poses as a critical signal transducer for EGFR with substantial synergistic effects believed to exist between these proteins [180]. When EGFR is activated by EGF, the RAS/RAF/MEK/ERK signaling pathway is initiated, stimulating the expression of cyclooxygenase-2 (COX-2) [181]. COX-2 is widely implicated to promote tumorigenesis as it is believed to promote angiogenesis, immune evasion, and anti-apoptotic activity [182]. **(Created with** BioRender.com**).**

Initially reported by Fritsche et al. in 2007, AhR was discovered as an important intracellular target for UVB [45]. AhR is expressed in all cutaneous cell types and contributes toward multiple physiological and pathophysiological processes of the skin [43]. Comprehensive reviews on AhR and skin cancer have been published [7,46,47]. AhR ligands, which can originate from both exogenously and endogenously are found in high concentrations within the skin. Solar UVB can generate AhR ligands from Trp in the skin, i.e., 6-formylindolo[3,2-b] carbazole (FICZ) [43,45]. The discovery of FICZ as a potent, endogenous ligand of AhR uncovers at least two signaling pathways initiated after UV-mediated AhR activation: one is the XRE-mediated gene transcriptional induction of CYP1A1, while the other is the AhR-activated EGFR internalization and subsequent induction of COX-2 [45,48-51] (Fig. 2). As both CYP1A1 and COX-2 play a critical role in tumor initiation and promotion, the UV-activated AhR contributes to photo-carcinogenesis [52]. Further studies have shown that the AhR pathway inhibited the repair of DNA damage by inhibiting nucleotide excision repair (NER) and that UV-induced skin cancer occurred less in the AhR knockout mice in comparison with the wild-type controls, further supporting the role of AhR in photo-carcinogenesis [53]. Using chemical and shRNA-mediated inhibition of AhR, AhR activation by UV via regulating E2F1 and checkpoint kinase 1, has been shown with the ability to inhibit apoptosis, which was confirmed in an AhR-knockout SKH-1 hairless mouse model [54]. Furthermore, the role of AhR in UV-mediated immunosuppression was demonstrated with AhR agonists and antagonists [55]. The AhR antagonist reduced UV-mediated immunosuppression and the induction of regulatory T (T_reg_) cells, while AhR agonist suppressed hypersensitivity reactions and induced antigen-specific T_regs_ similar to the UV. The role of AhR was further confirmed in AhR knockout mice in which UV-induced immunosuppression was significantly reduced [56]. Therefore, AhR activation by UVB-induced FICZ is believed to be the one important mechanism by which UVB causes skin cancer, by hampering DNA repair and apoptosis, and repressing the immune systems. Furthermore, based on animal and mechanistic studies, PAH and UV radiation may have synergistic or additive effects which could lead to enhanced effects inducing skin carcinogenesis [57,58]. The fact that both PAH and UV activate AhR further supports the notion that AhR pathway plays a central role in environmental factors induced carcinogenesis [46].

### 3.2. AhR in PAH-associated lung carcinogenesis

Lung cancer is an important health problem in the United States and worldwide, with the highest death rate and the lowest five-year survival rate. The primary factor that contributes to the development of lung cancer is tobacco smoking [59]. PAHs are a group of organic compounds produced during the process of incomplete combustion of various materials, such as coal and tobacco. Air pollution is another primary ambient source of PAHs for humans. AhR can be activated after inhalation of PAHs, which in turn triggers a series of events that promote the growth and spread of cancer [60,61]. The function of AhR in lung cancer has been recently reviewed [62,63]. PAHs have been linked to various cancers, including lung, skin, and breast cancer [46,52,64,65], while the susceptibility to cancer depends on the duration and the level of exposure.

**Fig. 3.**
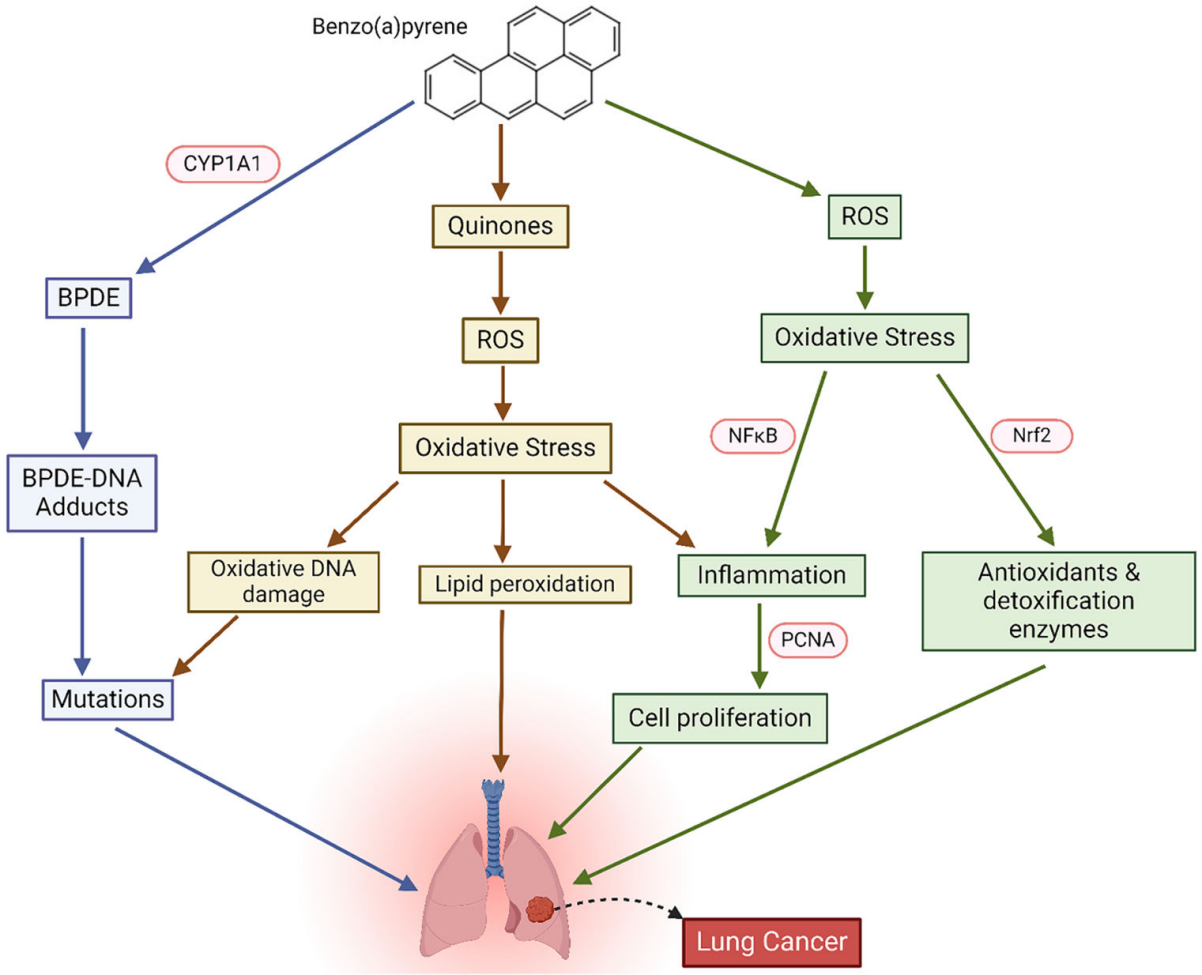
The mechanisms by which benzo(a)pyrene-induced carcinogenesis in the lungs occur can be assessed through multiple possible pathways. Benzo (a)pyrene (BaP) is metabolically activated through oxidation by CYP1A1, yielding the highly mutagenic and reactive metabolite, BPDE [155]. Production of B(a)P – *o* - quinones, B(a)P - radical species, and B(a)P – diol - epoxides, like BPDE, can create DNA damage by forming covalent BPDE-DNA adducts, contributing towards mutagenesis [55,66,67].Quinone compounds, converted from BaP by CYP1A1, produce ROS which causes oxidative stress-induced oxidative DNA damage as well as random lipid peroxidation, inflicting direct genomic instability [157,158,159]. Oxidative stress caused by elevated ROS levels activate NF-κB, stimulating a pulmonary inflammatory cascade by inducing transcription of proinflammatory genes [160]. Neutrophil cell proliferation at these sites of inflammation can occur due to high cytosolic PCNA levels expressed [162]. Oxidative stress-induced Nrf2 activation can also lead to greater proliferation and also anti-apoptotic gene expression, both attributes of lung carcinogenesis [163]. (Created with BioRender.com).

According to the International Agency for Research on Cancer (IARC), BaP, a PAH chemical found in cigarette smoke and polluted air, is classified as a Group 1 human carcinogen [66]. BaP has an impact on all three stages of carcinogenesis - initiation, promotion, and progression. The mechanisms by which BaP induces carcinogenesis can be divided into genotoxic and non-genotoxic mechanisms, by forming DNA damage and by binding to and activating AhR, respectively [67] (Fig. 3). The AhR-activated cytochrome P450 enzymes subsequently activate the pro-carcinogen BaP into active carcinogens such as BaP 7, 8 - diol 9, 10 – epoxide (BPDE). BPDE reacts with deoxyguanosine which forms a BPDE – N2 – dG adduct, promoting the formation of gene mutations in p53 [156]. The issue is exacerbated during failure to remove covalent BPDE-DNA adducts prior to DNA replication as DNA strand breakage and mutations due to chromosomal aberrations can occur, increasing lung cancer risk [155]. CYP1A1 and CYP1B1 are expressed in many cell types within the human respiratory system, including alveolar type I and II cells, ciliated columnar bronchoalveolar epithelial cells, and alveolar macrophages, with a higher level in smokers and ex-smokers than never-smokers [68,69]. CYP1A1 mediates the conversion of BaP into quinone compounds which may consequently produce ROS through redox reactions [157]. Imbalances between the generation of ROS and their elimination can cause oxidative stress to occur which can inflict damage towards DNA, regulate the progression of lung carcinogenesis, and alter signaling pathways intracellularly [158]. Oxidative stress as a result of elevated ROS levels enhances random lipid peroxidation, where its effects can manifest as alterations in cell membrane permeability and genomic instability, as well as oxidative stress-induced oxidative DNA damage [159]. Aside from BPDE-DNA adducts, oxidative stress-induced DNA damage grants direct routes to mutagenesis due to base modifications and abasic sites [187]. For example, the formation of 8 – oxo – 7, 8, - Dihydroguanine, as a result of guanine oxidization from ROS, confers mispairing with adenine through a conformational change during DNA replication [187]. Base mispairings can have deleterious effects in the form of truncating, nonsense, or missense mutations. Furthermore, activated AhR causes an inflammatory response that interrupts the immune system’s ability to function correctly, contributing to cancer [70,71]. AhR has been shown to be involved in the control of inflammatory responses within the respiratory tract through interaction with NF-κB as activation of this transcription factor can induce transcription of proinflammatory genes [160]. Neutrophils, an abundant leukocyte found at sites of inflammation, express high levels of cytosolic proliferating cell nuclear antigen (PCNA) which greatly correspond to increased cell proliferative activity, expectedly being that they are nuclear proteins involved in DNA synthesis and cell cycle regulation [162,161 72]. Additional to NF-κB, ROS-induced oxidative stress can activate the transcription factor Nrf2. In malignant cells, Nrf2 exhibits a pro-carcinogenic effect, given it enhances proliferation and anti-apoptotic gene expression, which have antioxidant response element-like sequences in their promotor regions [163]. This means that Nrf2, being a cellular protector, confers transformed cells defense against chemotherapy, enabling their progression and development. Moreover, activation of AhR with BaP stimulates MAPK signaling, which can affect cellular processes such as proliferation, differentiation, and apoptosis [73]. Oppositely, AhR has been shown to inhibit cigarette smoke-induced acute lung inflammation [74]. After exposure to cigarette smoke, AhR-deficient mice exhibit significantly higher levels of neutrophilia compared to the wild-type mice [75]. Additionally, the unbound form of AhR can associate with the NF-κB protein RelA in human lung cells, thereby influencing NF-κB activity and causing an increase in IL-6 expression [76]. Conversely, AhR activation can also facilitate the degradation of the RelA/p65 protein, both through the ubiquitin–proteasome system and via lysosomal degradation [77].

In addition to inducing classical AhR target genes such as CYP1A1, AhR has been shown playing a role in the regulation of immune checkpoints. BaP induced AhR-dependent expression programmed cell death 1 ligand (PD-L1) in lung epithelial cells [78]. Also, the endogenous ligand, kynurenine (kyn), stimulates programmed cell death 1 (PD-1) expression in cytotoxic T cells by activating AhR. After activation by PAH, AhR may play a role in inactivating the CD8^+^ T cells and inducing CXCL13 production [79]. PAH-induced lung cancer is attenuated in mice with the knockout of the CXCL13 gene. Therefore, AhR, activated by cigarette smoking, is involved in immunosuppression of lung tumors.

Furthermore, AhR influences the occurrence and development of cancer by regulating microRNA and small non-coding RNAs [80]. For example, MiR-196a is regulated by AhR in mouse lung fibroblasts. AhR can suppress the expression of MiR-96, which is closely related to the suppression of cancer development [81]. AhR plays an important role in the regulation of microRNAs expressed in the lungs as was observed in AhR knockout mice [62]. The expression of miR-22, miR-29a, miR-126a, and miR-193b was significantly increased in the lungs of male rats, while the level of miR-483 was increased in female rats exposed to BaP for a prolonged time. It has been suggested that the different roles of AhR in regulating microRNAs may affect the sex-dependent epigenetic inheritance of BaP [82]. Although evidence suggests that targeting AhR may help prevent and treat lung cancer, other studies indicate that AhR can act as a repressor of oncogenic signaling. AhR can act as a “double-edged sword” in lung carcinogenesis [62].

### 3.3. AhR in microbiota-associated colorectal cancer

**Fig. 4.**
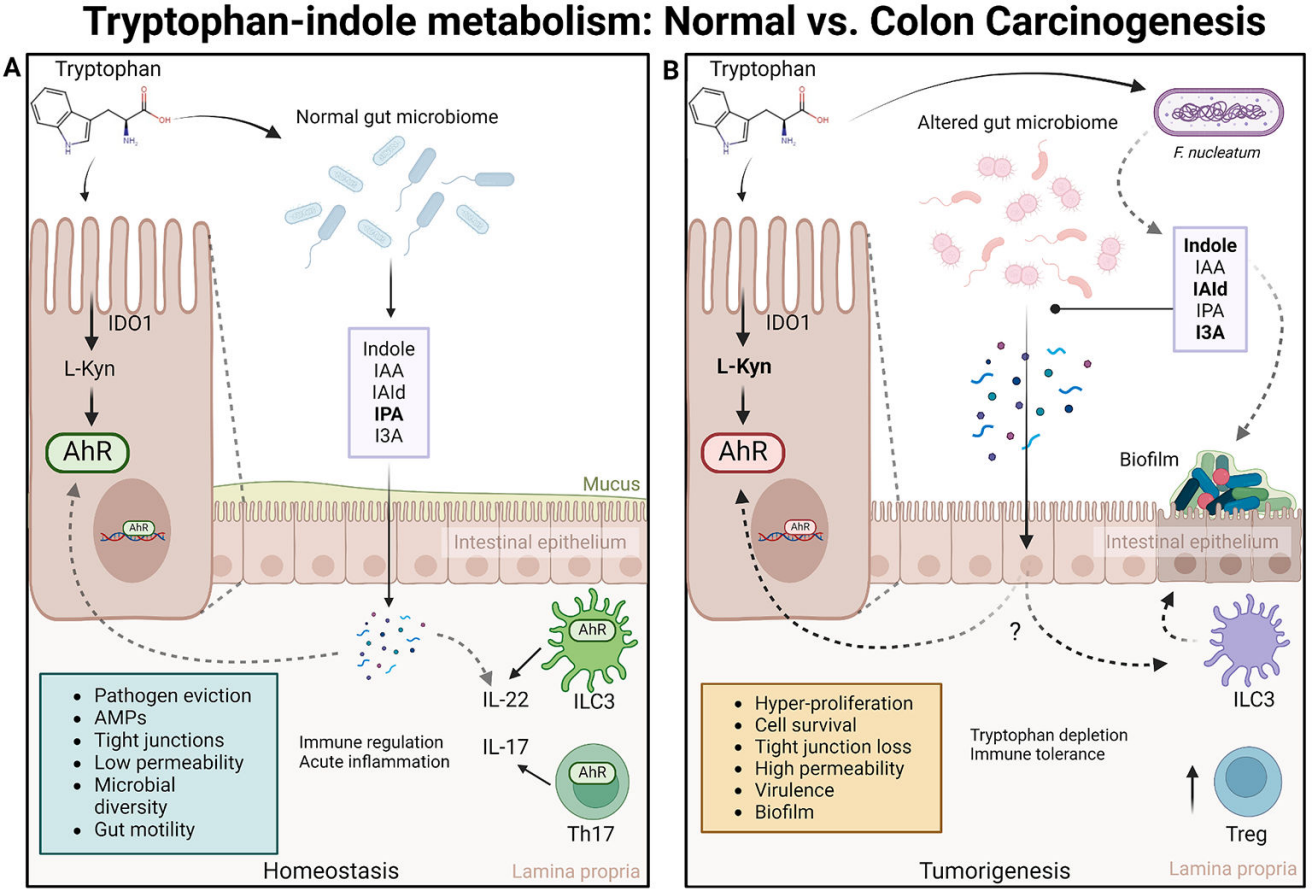
AhR-mediated regulation of intestinal homeostasis. a) Tryptophan is converted to L-kynurenine (L-Kyn) by Indoleamine 2,3-dioxygenase (IDO1) while a smaller percentage undergoes microbial metabolism [183]. Normal physiological conditions grant eviction of pathogens, promotion of antimicrobial peptides (AMPs), efficiency in tight junction protein activity, allowing higher selectivity for permeating ions, microbial diversity, and improved gut motility [183,184]. Microbiota in the gut, e.g., Bacteroides, Proteus vulgaris, Escherichia coli, convert tryptophan into indole and its derivatives by tryptophanase [185]. IPA is a significant metabolite in that it confers ROS-scavenging and anti-inflammatory effects on host health [186]. Commensal microbiota-derived indoles act via AhR, thus inducing IL-22 and IL-17 secretion, conferring enhanced immune and inflammatory regulation [187]. b) Overexpressed IDO1levels in epithelial cells result in the depletion of tryptophan, increasing L-Kyn levels, thus constitutive AhR activation [183]. Fusobacterium nucleatum promotes tumor development by stimulating immune responses and producing inflammatory factors through adhesion to the intestinal epithelium [188,189]. Fusobacterium nucleatum also mediates coaggregation of bacteria to form biofilms, causing depletion of mucous on the intestinal barrier, thus increased gut permeability and mucosal inflammation [190]. Stronger AhR activation may also induce greater T_reg_ cell activation, favoring an immunotolerant environment also [183]. It is still not fully understood how microbial indolic metabolites influence immune or epithelial cell metabolism in colon cancer. (Created with BioRender.com).

The human GI tract has one of the largest interfaces (250 ~ 400 m^2^) between the host and the external environment of the body [83]. In the GI tract, AhR plays a pivotal role in regulating immunity and maintaining intestinal homeostasis [84-89] (Fig. 4). AhR-mediated regulation of intestinal homeostasis can occur through two main mechanisms: 1) by promoting an anti-inflammatory response through the activation of T_reg_ cells; 2) by enhancing intestinal mucosal integrity via the activation of Th17 cells and induction of IL-22 [84,90-92]. Any disruption in AhR expression or activity can lead to altered intestinal homeostasis and potentially contribute to carcinogenesis (for review, see [83,93]).

Colorectal cancers rank third in cancer incidence and cancer-related deaths for both men and women [94]. Various endogenous ligands can activate AhR, including Trp metabolites, dietary compounds, microbial Trp metabolites, bilirubin, arachidonic acid, prostaglandins, and cytokines. Kawajiri et al. [95] were the first to report that global deletion of the AhR promotes intestinal carcinogenesis and that the natural AhR ligands, such as indole-3-carbinol (I3C) significantly reduced tumor burden in ApcMin/+ mice, but not in ApcMin/+AhR−/− mice [95]. Consistently, whole body AhR deletion enhances colitis-associated colorectal tumorigenesis, and supplementation of I3C reduces the number of colorectal tumors in WT, but not in AhR-null mice [96]. Recent evidence indicates intestinal-specific deletion of the AhR promotes carcinogen-induced and colitis-associated colon tumorigenesis [97,98].

The gut microbiota comprises a vast array of microorganisms that engage in continuous two-way communication with the host, partially facilitated by the production of diverse bacterial metabolites. The host and bacterial metabolism of Trp provides a robust and consistent pool of AhR ligands that regulate systemic AhR activity and contribute toward homeostatic maintenance [99]. The changes in Trp metabolism commence early in colon carcinogenesis and enable immune evasion within the tumor microenvironment. Elevated levels of Kyn in the tumor microenvironment play a crucial role in promoting immune evasion and survival as it drives constitutive AhR activation which enhances the transcription of genes necessary for cancer cell proliferation and tumor escape, and metastasis of tumors through T cell inactivation [98-102,188]. This could deem elevated levels of Kyn a tumor-adaptive mechanism in the gut. Stronger AhR activation may also induce the over-production of IL-22 and IL-17 and Treg cell activation, conferring an immunotolerant environment [188]. In malignant tumors, increases in T_reg_ cells can obstruct effective anti-tumor immune responses and IL-22 secreted by ILC3, contributing towards tumor growth. Thus, the Kyn-AhR signaling pathway contributes to colon cancer progression [86,100-103].

Recently, an intestinal microbiota-derived short-chain fatty acid (SCFA) known as butyrate has gained increasing attention. Butyrate constitutes approximately 15–23 % of the total SCFA in human feces, ranging from 10 to 25 mM [104]. The role of butyrate in the development of colorectal cancer remains a subject of debate. Some studies suggest that butyrate might play a role in preventing colorectal cancer because it exhibits histone deacetylase (HDAC) inhibitory activity and can promote cell cycle arrest, differentiation, and apoptosis in colorectal cancer cells at physiological concentrations [105]. Supporting this notion, Zagato et al.[106] provided evidence of butyrate’s tumor suppressor effect. Conversely, Belcheva et al. proposed that butyrate may promote the development of colorectal cancers [107]. At lower concentrations, butyrate reduces the expression and activity of CYP1A1, leading to decreased clearance of indole metabolites, e.g., FICZ, and increased access of these metabolites to the lamina propria. This, in turn, stimulates intestinal immunity by promoting the secretion of IL-22 and IL-17 from innate lymphoid cells (ILC3) and Th17 cells, respectively. These cytokines contribute to enhanced production of mucin and antimicrobial peptides, leading to improved intestinal barrier function. On the contrary, higher concentrations of butyrate have been found to augment CYP1A1 expression and activity through HDAC inhibition, resulting in increased clearance of indole metabolites and reduced entry into the lamina propria [108]. Via modulating histone acetylation, butyrate induces CYP1A1 expression which alters BaP metabolism in colon epithelial cell lines [109]. Consequently, butyrate contributes to the tolerance of commensal bacteria by upregulating the expression of AhR and IL-10 receptors, as well as promoting the differentiation and expansion of intestinal IL-10-releasing T_reg_ and Tr1 cells. Moreover, recent findings indicate that butyrate, along with propionate and valerate, can directly bind to AhR at concentrations ≥ 1 mM, independently of HDAC inhibition, thereby activating its signaling pathway and increasing CYP1A1 expression in human intestinal cell lines [110]. These findings are particularly relevant for patients with inflammatory bowel disease (IBD) as they often exhibit lower colonic SCFA concentrations and a higher proportion of butyrate-producing bacteria. The role of AhR in carcinogenesis is complex since AhR’s ability to bind to various ligand types leads to either the enhancement or suppression of carcinogenesis [8,95,96,98,111,112]. Both AhR agonists and antagonists may have potential cancer-preventive activity. The ability of AhR to bind to various endogenous and nonendogenous ligands emphasizes the intricacies of its involvement in carcinogenesis of the colon. This is reflected upon the fact it encompasses both tumor-suppressive and -promoting characteristics, depending on a plethora of factors identified from the studies aforementioned [8,95,96,98,111,112]. Due to this, it is necessary to further research amongst both the agonists and antagonists of AhR to improve our understanding of their effects in colon cancer, given the potential of AhR as a target of interest in cancer treatment and prevention.

## 4. Immunological studies of AhR

Historically, studies of AhR have centered upon its capacity to function as a receptor for environmental toxins, like TCDD and BaP, and endogenous ligands, such as FICZ, 2-(1′H-indole-3′-carbonyl)-thiazole-4-carboxylic acid methyl ester (ITE), and Kyn [113-116]. However, the field has more recently expanded to investigate the functions of AhR in the context of immunity. AhR is reportedly involved in various immune cell developments and functions, which can help to further understand its role in carcinogenesis (for review, see [117-120]).

AhR activation can help to enhance antitumor immunity. For example, natural killer (NK) cells are amongst the main components of the innate immune system and are critical in antitumor immune responses. Using in vivo experiments, Shin et al. demonstrated that the cytolytic activity and capacity of NK cells to suppress tumor formation in RMA-S, an MHC class I-deficient mouse lymphoma cell line, is impaired in the absence of AhR [121]. In contrast, AhR activation induced by the endogenous ligand, FICZ, can potentiate NK cells to increase IFN-γ secretion while simultaneously enhancing cytolytic activity and antitumor activity [122]. The effects on NK cells can result in enhanced antigen presentation, macrophage activation, and antiviral and antibacterial immunity. In another study, by Wagage et al., it has been suggested that the production of IL-10, an anti-inflammatory cytokine, requires AhR activation in NK cells, indicating the importance of AhR in preventing inflammation by indirectly down-regulating pro-inflammatory cytokines. They experimented with isolated NK cells from *Toxoplasma gondii*-infected AhR (−/−) mice and found that IL-10 secretion was impeded due to increased resistance to such infection [123]. Aberrant expression of IL-10 can result in increased inflammatory responses to microbes as they cannot downregulate the secretion of pro-inflammatory cytokines proficiently. A third study that demonstrates the importance of AhR in NK cells showed that the NK cells in the liver constitutively express AhR, and in AhR (−/−) mice, AhR deficiency resulted in greater susceptibility to cell death induced by cytokines [124]. This is a reasonable notion given AhR is a sensor for many exogenous stimuli and has the ability to combine signals from toxins, dietary metabolites, and more, into the immune response. The role of AhR in NK cells-mediated immune surveillance needs further investigation.

AhR also plays an important role in the impairment of white blood cell development following its activation, furthering our knowledge of its involvement in the immune response. Environmental AhR ligands like PAHs and dioxins suppress immunity by compromising lymphocyte development, activation, and effector function [125]. For example, the high-affinity AhR ligand, TCDD, demonstrated its immunosuppressive abilities by inhibiting lymphocyte development in vivo significantly, even at doses ranging from 10 to 100 ng/kg [126,127]. Administration of this dose resulted in significant increases in mortality following influenza virus infection, as well as memory responses being weakened [126-131]. Furthermore, TCDD at slightly higher doses, 100 – 1,000 ng/kg), induced thymic atrophy [126,132-134], suppressed humoral responses in an AhR-dependent manner [135,136], and lowered resistance to parasites [137].

In contrast, AhR may also be involved in facilitating tumor immune escape. In the tumor microenvironment, immunosuppressive effects are mediated by Th17 cells, type-1 T_reg_ cells (Tr1), and thymus-derived T_reg_ cells, all suppressing the proliferation and cytokine secretion of effector cells [138]. AhR can instigate the production of Th17 cells and T_reg_ cells based on the concentration of TCDD in experimental autoimmune encephalomyelitis (EAE), suggesting that the distribution between Th17 and T_reg_ cells poses an important role in autoimmune disease [139]. Based on the immunosuppressive effects of AhR ligands on autoimmune diseases, such as EAE, it would be reasonable to propound that activation of AhR in the tumor microenvironment corresponds to increased numbers of T_reg_ cells, which could also account for the tumor-promoting properties of TCDD [122]. It was suggested by Opitz et al. that Kyn had a role in the proliferation of CD4^+^ and CD8^+^ T cells, depending on concentration, and they affirmed that through the AhR, Kyn suppressed antitumor immune responses in sections of human gliomas [140]. AhR is required in T cells for optimal production of Foxp3^+^-generating T_reg_ cells and it is Kyn that stimulates the production of these cells through AhR [141]. The presence of AhR is also important in enabling the formation of Tr1 cells in humans and mice, which inhibits autoimmune responses by interacting with transcriptional factor macrophage-activating factor to enhance expression of the cytokines that help in immune response regulation, namely, IL-10, IL-21, and IL-27 [122,142].

Therefore, AhR activation appears to have a dual role in promoting antitumor immunity and facilitating tumor immune escape, depending on which type of immune cells are involved. For applications in cancer prevention, future studies may investigate the targeted delivery of AhR agonists or antagonists to specific immune cells.

## 5. Example AhR agonists, antagonists, and their efficacy in cancer chemoprevention

Multiple chemicals with diverse structures have been evaluated as AhR ligands. Many of these chemicals are derived from natural products, for example, flavonoids, with flavonol quercetin as a prototypical representative (for review, see [4]). Some studies suggest that activating the AhR by specific ligands promotes cancer [143], while others have shown that the AhR activation protects against the effects of environmental carcinogens [142]. However, many of the agents have AhR independent effects. Future studies should focus on the evaluation of AhR specific modulators. Here we briefly review several examples.

### 5.1. Proton pump inhibitors (PPIs)

The PPI omeprazole has been shown as an AhR agonist [144-146], since it induces CYP1A1 and exhibits other agonist activities. Omeprazole demonstrated activity in suppressing proliferation, migration, invasion, and cell cycles of esophageal squamous cell carcinoma, which are AhR-dependent [147]. The same effects were observed in preclinical studies of glioma [148], pancreatic cancer [149,150], breast cancer [151]. In vitro experiments using MDA-MB-231 breast cancer cells showed that omeprazole decreased the expression of two genes associated with promoting metastasis, namely, matrix metalloproteinase-9 (MMP-9) and C-X-C chemokine receptor 4 (CXCR4). Further analysis revealed that the downregulation of CXCR4 by omeprazole was dependent on the activation of AhR [151]. Omeprazole is a non-traditional activator of AhR as it regulates AhR action through phosphorylation rather than binding to the receptor [152]. Omeprazole did not alter CYP1A1 mRNA and protein levels induced by B(a)P, but inhibited CYP1A1 enzyme activity [153]. Unlike typical AhR activators, omeprazole has shown no experimental evidence of carcinogenic activity. Chronic administration of omeprazole did not show effects promoting esophageal carcinogenesis [154]. Instead, omeprazole demonstrated chemopreventive activity against carcinogen-induced colonic adenoma in azoxymethane-induced rat colorectal cancer model [155-157]. However, it is unknown whether the cancer chemopreventive effects of omeprazole is dependent on AhR activation.

### 5.2. Metformin

Belonging to the biguanide class of drugs, metformin is an oral antidiabetic drug traditionally used for the treatment of type 2 diabetes mellitus [158]. Amongst its several mechanisms of action, one of the ways in which metformin exerts its effects is by activating adenosine monophosphate kinase (AMPK), triggering the inhibition of enzymes essential for gluconeogenesis and glycogen synthesis within the liver, while simultaneously inducing insulin signaling and glucose transportation in muscles [158]. Metformin also serves to be an excellent example of drug repurposing, given that it encompasses profound chemopreventive qualities. In a study by Do et al., metformin down-regulated constitutive and inducible CYP1A1 and CYP1B1 expression in breast cancer cells [159,160]. Do et al. performed a series of Western blots to assess CYP1A1 and CYP1B1 protein levels in MCF-7, a human breast cancer cell line, following treatment of metformin (1–5 mM) for 24 h. Results subsequently showed that metformin down-regulated enzymatic protein levels in a dose-dependent manner and expression of the two enzymes were also inhibited in a time-dependent manner [159]. Also, CYP1A1 and CYP1B1 protein levels were stoutly increased upon TCDD treatment as expected, however, the CYP1A1 and CYP1B1 induction induced by this carcinogen were notably eradicated by metformin pre-treatment in MCF-7 cells [159]. In vivo, metformin has shown chemopreventive effects on DMBA-induced breast carcinogenesis in rat model by inhibiting CYP1A1/CYP1B1 [161].

### 5.3. Resveratrol

Found in red wines and a plethora of human foods, resveratrol is a natural polyphenolic phytoalexin synthesized by plants undergoing infectious or ionizing radiation [162]. Various bioactivities of resveratrol have been associated with health enhancement. For example, its polyphenolic structure confers antioxidant effects which have been linked to reduced free radical-induced apoptosis and low-density lipoprotein oxidation, whereas its cardiovascular protective effects have been suggested through its ability to inhibit platelet aggregation and the promotion of artery vasorelaxation [162]. It is predominantly the *trans* isomeric form of resveratrol for which its cellular induction responses and anti-cancer activities are attributed to [162]. Resveratrol is a competitive antagonist of dioxin and other AhR ligands, such as BaP [163]. Resveratrol binds to the AhR, evoking nuclear translocation however, it inhibits the induction of transcription driven by dioxin-responsive elements [164]. Aside from resveratrol’s chemopreventive properties, its chemotherapeutic properties have been associated with its pro-apoptosis, antioxidant, anti-inflammatory, and anti-proliferative effects [162,165,166]. For instance, Li et al. examined these anti-proliferative and apoptotic effects in HeLa cervical carcinoma cells and found that resveratrol had induced cell shrinkage and apoptosis through activation of caspase-3 and –9 and increased p53 expression, a protein vital for cell cycle progression and cell survival [162,167]. Furthermore, resveratrol displayed much competence in use for combination therapy, notably in breast cancer [162,168]. The phytoalexin has demonstrated its ability to reverse drug resistance in a range of cell systems, in vitro, by sensitizing tumor cells to effects mediated by drugs in combination with other chemotherapeutic agents [162,165]. Outstanding resveratrol research has shown profound results so far, making it a promising candidate for the treatment and prevention of several cancers.

### 5.4. Curcumin

A further example of the repurposing of compounds lies within a natural polyphenol found in the rhizomes of turmeric, curcumin. Curcumin is a diarylheptanoid, part of the curcuminoids group, which are phenolic pigments and secondary metabolites of turmeric, mainly used as coloring agents and dietary constituents. Additional to this is curcumin’s therapeutic role as it has been shown to display antitumor effects and has been identified as a ligand for the AhR [169]. The interaction of curcumin with the AhR pathway was examined by Ciolino et al. in MCF-7 mammary epithelial carcinoma cells [170]. It was found that curcumin activated the DNA-binding capacity of the AhR for the dioxin-responsive element of CYP1A1, as measured by using the electrophoretic-mobility shift assay, a means of assessing whether a protein can interact directly with a small DNA sequence [170]. In this study, curcumin was able to compete with prototypical AhR ligand, TCDD, for AhR binding within the cytosol of isolated MCF-7, implying that curcumin directly interplays with the receptor [170]. Using RT-PCR, CYP1A1 was measured in mammary carcinoma cells that were treated prior with 1 μM of DMBA in both the absence and presence of curcumin and after 24 h, a 15-fold increase in CYP1A1 mRNA was observed [170]. One could deem curcumin a partial antagonist as a 5 μM treatment partially, and competitively, inhibited the increase caused by DMBA [170]. Moreover, the diarylheptanoid stifled DMBA metabolic activation and even decreased toxicity induced by DMBA too [170].

### 5.5. β-blockers

Propranolol is a non-selective β-blocker, prescribed for controlling hypertension. Propranolol has been reported with ability to activate AhR [171]. Exposure to UV light strongly increased the capacity of propranolol to activate the AhR and induced a proinflammatory AhR signaling pathway, identical to that induced by the UV light-induced FICZ. Activation of AhR by UV and propranolol may explain their inducting effects of lupus erythematosus. AhR can be upregulated by propranolol treatment in tumor tissue, which leads to the inhibition of Ki-67, a pro-metastatic biomarker. The administration of the drug may be advantageous for breast cancer patients as it can potentially decrease systemic inflammation and suppress the expression of biomarkers associated with metastasis and tumor growth. Thus, propranolol exhibited a positive impact on biomarkers associated with metastasis and inflammation in excised tumor tissue.

Another non-selective β-blocker carvedilol has been reported with ability to antagonizing BaP-induced AhR activation [172]. Carvedilol effectively prevented the malignant transformation of human bronchial epithelial cells induced by BaP. Additionally, carvedilol inhibited the activation of oncogenic signaling pathways, namely ELK-1 and NF-κB, which were induced by BaP exposure. These findings indicate that the interplay between these signaling pathways plays a significant role in mediating both inflammation and the development of cancer in the lung.

## 6. Conclusions and future direction

AhR plays an important role in the developing several common types of cancer that originate from barrier organs, skin, lung, and gut. Numerous in vitro and in vivo evidence has shown that AhR plays a crucial role in mediating the toxic effects of environmental carcinogens, such as UV radiation and PAHs. However, AhR is involved in carcinogenesis with both pro- and anti-tumor functions in different types of cancer, which possibly means that specific ligands may drive or suppress carcinogenesis. The exact functions of AhR signaling during cancer development are still under investigation, and more studies are required to fully understand its role in the carcinogenesis. Although some preclinical studies demonstrated the efficacy of AhR agonists and antagonists on carcinogenesis models, future studies should evaluate specific agonists and antagonists.

## Abbreviations:

AhR: aryl hydrocarbon receptor
ARNT: AhR nuclear translocator
BaP: benzo(a)pyrene
CML: chronic myeloid leukemia
CXCR4: C-X-C chemokine receptor 4
FICZ: 6-formylindolo[3, 2-b]carbazole
GI: gastrointestinal
GST: glutathione S-transferase
Hsp90: heat – shock protein 90
IARC: International Agency for Research on Cancer
Kyn: kynurenine
HDAC: histone deacetylase
ILC3: innate lymphoid cells
I3C: indole-3-carbinol
IBD: inflammatory bowel disease
NQO1: NAD(P)H quinone dehydrogenase 1
NK: natural killer
EAE: experimental autoimmune encephalomyelitis
PAH: polycyclic aromatic hydrocarbon
PD-L1: programmed cell death 1 ligand
PD-1: programmed cell death 1
PM: particulate matter
SCC: squamous cell carcinoma
SCFA: short-chain fatty acids
TCDD, 2, 3, 7, 8: tetrachlorodibenzodioxin
T_reg_: regulatory T cells
Trp: tryptophan
UV: ultraviolet
XRE: xenobiotic-responsive element.
